# CMT-linked loss-of-function mutations in *GDAP1* impair store-operated Ca^2+^ entry-stimulated respiration

**DOI:** 10.1038/srep42993

**Published:** 2017-02-21

**Authors:** Paloma González-Sánchez, David Pla-Martín, Paula Martínez-Valero, Carlos B. Rueda, Eduardo Calpena, Araceli del Arco, Francesc Palau, Jorgina Satrústegui

**Affiliations:** 1 Departamento de Biología Molecular, Centro de Biología Molecular Severo Ochoa, Consejo Superior de Investigaciones Científicas–Universidad Autónoma de Madrid (CSIC-UAM), Madrid, 28049, Spain; 2 Centro de Investigación Biomédica en Red de Enfermedades Raras (CIBERER), Madrid, 28029, Spain; 3 Instituto de Investigación Sanitaria Fundación Jiménez Díaz, IIS-FJD, Madrid, 28040, Spain; 4 Program in Rare and Genetic Diseases and IBV/CSIC Associated Unit, Centro de Investigación Príncipe Felipe, Valencia, 46012, Spain; 5 Facultad de Ciencias Ambientales y Bioquímica, Universidad de Castilla la Mancha, Toledo, 45071, Spain; 6 Institut de Recerca Sant Joan de Déu and Hospital Sant Joan de Déu, Barcelona 08950, Spain; 7 Pediatrics Division, University of Barcelona School of Medicine, Barcelona, Spain

## Abstract

GDAP1 is an outer mitochondrial membrane protein involved in Charcot-Marie-Tooth (CMT) disease. Lack of GDAP1 gives rise to altered mitochondrial networks and endoplasmic reticulum (ER)-mitochondrial interactions resulting in a decreased ER-Ca^2+^ levels along with a defect on store-operated calcium entry (SOCE) related to a misallocation of mitochondria to subplasmalemmal sites. The defect on SOCE is mimicked by MCU silencing or mitochondrial depolarization, which prevent mitochondrial calcium uptake. Ca^2+^ release from de ER and Ca^2+^ inflow through SOCE in neuroblastoma cells result in a Ca^2+^-dependent upregulation of respiration which is blunted in GDAP1 silenced cells. Reduced SOCE in cells with CMT recessive missense mutations in the α-loop of GDAP1, but not dominant mutations, was associated with smaller SOCE-stimulated respiration. These cases of GDAP1 deficiency also resulted in a decreased ER-Ca^2+^ levels which may have pathological implications. The results suggest that CMT neurons may be under energetic constraints upon stimulation by Ca^2+^ mobilization agonists and point to a potential role of perturbed mitochondria-ER interaction related to energy metabolism in forms of CMT caused by some of the recessive or null mutations of GDAP1.

Charcot-Marie-Tooth (CMT) disease is the most common inherited neuromuscular disorder characterized by wide locus heterogeneity^1^,^2^. Mutations in the *GDAP1* gene show phenotypic and Mendelian heterogeneity in CMT patients and lead to several forms of CMT including recessive demyelinating (CMT4A)^3^, recessive axonal (AR-CMT2K)^4^, recessive with intermediate clinical features (CMTRIA)^5^ and a dominant inheritance pattern and axonal features (CMT2K)^6^,^7^.

GDAP1 is an outer mitochondrial membrane protein containing glutathione-S-transferase type domains^8^, and it has been related to mitochondrial fission/fusion^9^,^10^,^11^,^12^ or redox processes^13^,^14^. On the other hand, GDAP1 interacts with caytaxin and RAB6B, involved in anterograde-retrograde movement of vesicles^15^. Given the strategic localization of GDAP1 in the outer mitochondrial membrane, and the number of interacting partners of the protein, it is expected that mutations in the protein can give rise to numerous nonexclusive effects on cell function, and understanding which of them is ultimately responsible for the disease phenotype is a real challenge.

*GDAP1*-knockdown (KD) in the human neuroblastoma SH-SY5Y cells results in a defect in store-operated calcium entry (SOCE)^15^, a calcium entry pathway activated after discharge of ER-Ca^2+^ stores^16^. SOCE is regulated by mitochondria in different cell types^17^,^18^,^19^,^20^, and Ca^2+^ uptake by mitochondria through mitochondrial calcium uniporter (MCU) regulates both STIM1 activation and SOCE maintenance by preventing its Ca^2+^-dependent slow inactivation^19^,^20^. *GDAP1*-KD cells and motoneurons from *Gdap1*-KO mice^21^ have a reduced SOCE activity, associated with reduced SOCE-driven calcium uptake in mitochondria. This was not due to an intrinsic defect in mitochondrial calcium uptake but to a misallocation of mitochondria close to the subplasmalemmal sites^15^. Reduced SOCE activity in *GDAP1*-KD cells was attributed to a Ca^2+^ induced inactivation of SOCE due to the lack of Ca^2+^ uptake by nearby mitochondria^15^. Junctophilin-1 (JPH1) protein, encoded by a *GDAP1* gene modifier, plays a role in Ca^2+^ homeostasis, and is able to restore SOCE activity in *GDAP1*-KD cells. The presence of mutations in both genes (*JPH1* and *GDAP1*) has been associated with a more severe phenotype^22^.

The role of SOCE has been related to ER-Ca^2+^ refilling^23^,^24^. In hippocampal neurons, SOCE has been shown to be necessary for maintaining ER-Ca^2+^ levels, which are continuously lost at rest^23^. We have observed that *GDAP1*-KD or *Gdap1*-KO cells have lower ER-Ca^2+^ levels^21^,^22^ associated with the defect in SOCE.

The aim of the present study is to explore the functional consequences of impaired SOCE activity in mitochondrial function of GDAP1 deficient cells. Recent findings have revealed new roles of calcium and its pathway-specific interaction with mitochondria in bioenergetics. For example, basal respiration decreases in cells lacking MCUR1^25^, a putative regulator of the MCU (but see ref. 26), in cells with lower mitochondrial Ca^2+^ transients upon stimulation^25^, or in cells lacking IP_3_ receptors or incubated with antagonists of IP_3_ receptors in which the lack of matrix Ca^2+^ stimulates autophagy^27^,^28^. In addition, workload-induced stimulation of respiration depends upon Ca^2+^ signaling in mitochondria, as shown in neurons lacking Ca^2+^-regulated AGC1/Aralar, a Ca^2+^-dependent component of the malate aspartate shuttle^29^,^30^, and cardiomyocytes lacking MCU^31^,^32^, but not in other studies^33^. This raises the possibility that reduced SOCE activity from GDAP1 deficiency impacts on neuronal respiration and thereby may influence ATP production in the affected neurons. Therefore, we have explored this possibility in neuroblastoma. In addition, we have studied the effects of *GDAP1* pathological missense mutations in SOCE activity and SOCE-induced stimulation of respiration.

The results show that GDAP1 deficiency results in a defect of SOCE activity and ER- Ca^2+^ levels, with a decrease in SOCE-stimulated respiration which is reproduced by recessive mutations located in the α-loop region of GDAP1 involved in mitochondrial movement, but not by other mutations. The specificity of these defects for different mutants may aid in understanding the pathogenic mechanisms of CMT.

## Results and Discussion

### Ca^2+^ signaling is required to upregulate respiration in response to SOCE

**Figure 1:**
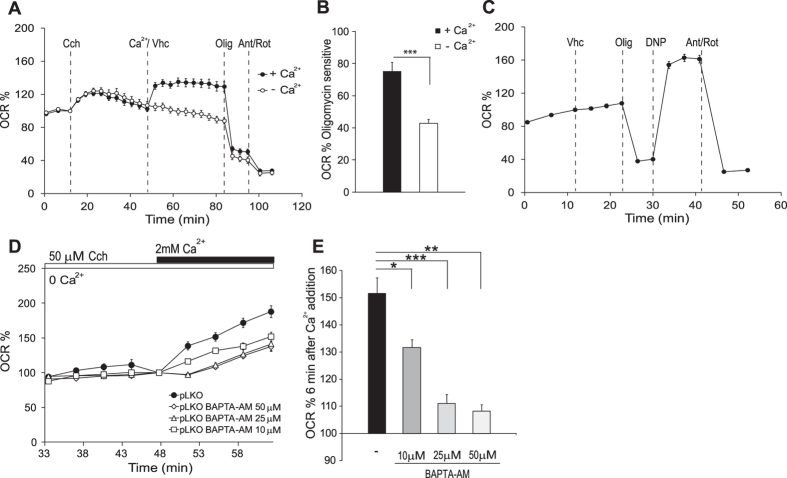
Stimulation of respiration induced by SOCE depends on Ca^2+^ signaling. (**A**) Oxygen consumption rate expressed as percentage of basal OCR in control pLKO cells showing the sequential injection of carbachol (Cch, 50 μM), vehicle (Veh) or Ca^2+^ (2 mM) and metabolic inhibitors: oligomycin (Olig, 6 μM) and antimycin A/rotenone (Ant/Rot, 1 μM/1 μM) at the indicated time points. (**B**) Quantification of oligomycin sensitive OCR expressed as percentage of basal OCR in control pLKO cells. The effect of calcium was significant (n = 27, obtained from at least 8 independent experiments). (**C**) Oxygen consumption rate expressed as percentage of basal OCR in control pLKO cells at the time of vehicle addition. Sequential injection: vehicle, oligomycin, DNP (0.25 mM) and antimycin A/rotenone at the indicated time points. (**D**) SOCE-stimulation of respiration in absence or presence of BAPTA-AM (50, 25 or 10 μM). Oxygen consumption rate expressed as percentage of OCR after carbachol addition (Cch, 50 μM) in Ca^2+^-free medium. (**E**) Quantification of % OCR 6 min after calcium addition, (n = 3–12, from at least 3 independent experiments). All data are expressed as mean ± SEM. Means were compared using one-way and two-way ANOVA, *p < 0.05, **p < 0.01, ***p < 0.001, *posthoc* Bonferroni test.

Neuroblastoma SH-SY5Y cell line can experience substantial Ca^2+^ influx through SOCE channels^15^. The role of Ca^2+^ in tuning ATP production to ATP demand in excitable cells has been known for a long time^34^,^35^,^36^,^37^, and recently, Ca^2+^ has been shown to cooperate in adjusting coupled respiration to ATP demand under the workloads induced by carbachol, high K^+^ depolarization or veratridine in neurons^29^,^30^,^38^. We analyzed whether SOCE-driven Ca^2+^ signals stimulate mitochondrial respiration in control SH-SY5Y pLKO neuroblastoma cells previously described^15^. To this end, SOCE was activated by carbachol, which mobilizes ER-Ca^2+^ through activation of IP_3_ receptors, followed by the addition of 2 mM CaCl_2_. Ca^2+^ strongly stimulated respiration (Fig. 1A), mainly coupled respiration, as it was largely inhibited by oligomycin (Fig. 1B). In the absence of external Ca^2+^ (vehicle), the increase in oxygen consumption rate (OCR) was not observed (Fig. 1A). SOCE-induced stimulation of respiration was about 140% of initial values, smaller than the maximal respiration obtained after uncoupler addition (Fig. 1C).

We next studied whether SOCE-stimulated respiration was due to an increase in ATP demand or through a direct effect of cytosolic Ca^2+^ on oxidative phosphorylation. To this end, control neuroblastoma cells were preincubated with different concentrations of BAPTA-AM, a rapid intracellular Ca^2+^ chelator^39^ before measuring SOCE-stimulated respiration. BAPTA loading prevents cytosolic Ca^2+^ signals but does not change Ca^2+^ inflow through SOCE channels and thereby maintains the SOCE-induced workload^29^. In BAPTA-AM (50 and 25 μM) loaded cells Ca^2+^ stimulation of respiration was abolished during the first 3 min after Ca^2+^ readmission (Fig. 1D), and the stimulation observed thereafter was much lower in the presence of the chelator than in its absence. A low chelator concentration (10 μM) had smaller, yet significant effects on Ca^2+^ stimulation respiration (Fig. 1D,E). Therefore, the results indicate that Ca^2+^ signaling itself through regulation of mitochondrial respiration is required to couple respiration to SOCE activity.

### Mitochondrial Ca^2+^ uptake regulates SOCE activity in neuroblastoma cells

**Figure 2:**
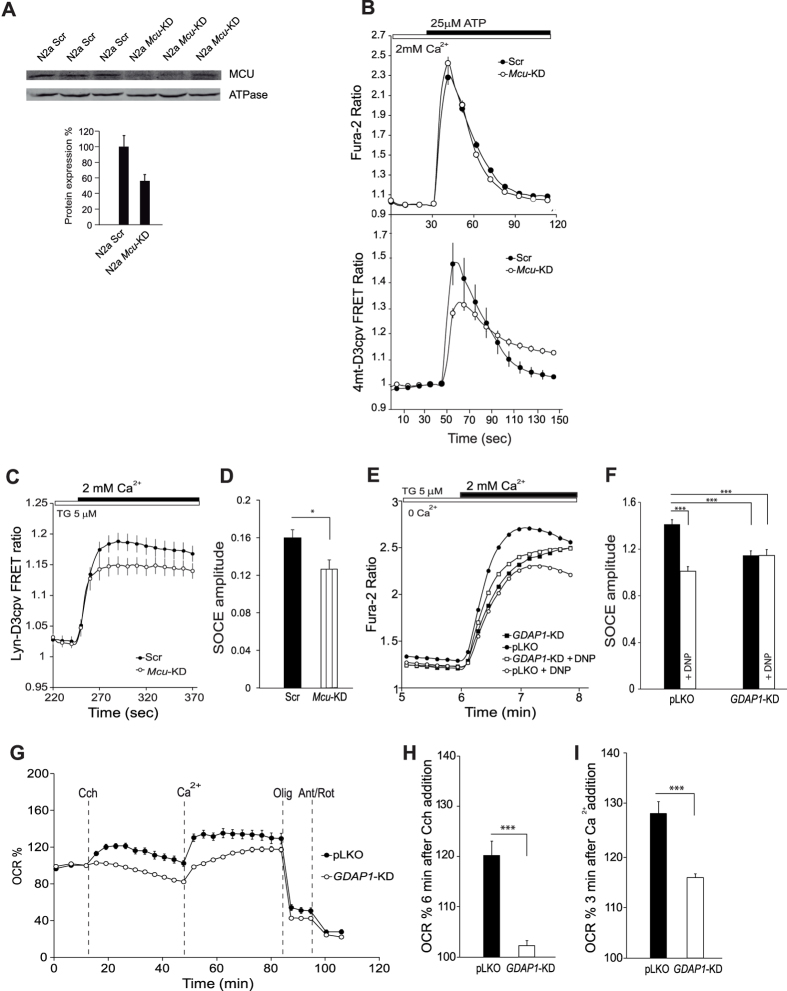
Mitochondrial Ca^2+^ uptake during SOCE and SOCE-stimulation of respiration is reduced in GDAP1-KD cells. (**A**) Analysis of MCU levels by Western blot. Protein extracts were obtained 72 hours after transfection of N2a cells with either shScr or shMcu. Primary antibodies used were α-MCU and α-βATPase as a control. MCU protein levels drop to 56, 2 ± 8, 3% of control values. (**B**) Fura-2 [Ca^2+^]_i_ signals and 4mt-D3cpv mitochondrial calcium signals in N2a cells transfected with shScr or shMcu upon addition of 25 μM ATP where indicated. (**C**) Lyn-D3cpv subplasmalemmal Ca^2+^ signals were measured in N2a cells transfected with shScr or shMcu upon addition of 2 mM Ca^2+^ in Ca^2+^-free medium with 5 μM Thapsigargin (Tg). Data were obtained from 3 independent experiments (n = 9–16 cells). (**D)** Quantification of SOCE amplitude as ΔRatio (F510/F440) ± SEM for each condition. (**E)** SOCE response in control pLKO and *GDAP1*-KD neuroblastoma cells, in presence or absence of DNP (0.25 mM). Fura-2 [Ca^2+^]_i_ signals were measured upon addition of 5 μM Tg in Ca^2+^ -free medium and 2 mM CaCl_2_ where indicated. DNP was added 2 min before Ca^2+^ addition. Traces were obtained averaging at least 250 cells from at least 4 independent experiments. (**F)** Quantification of SOCE amplitude as ΔRatio (F340/F380) ± SEM for each cell line and condition. (**G)** Oxygen consumption rate expressed as percentage of basal OCR in control pLKO and *GDAP1*-KD cells, showing the sequential injection of carbachol (Cch, 50 μM), Ca^2+^ (2 mM) and metabolic inhibitors. (**H**,**I)** Quantification of % OCR 6 min after carbachol addition and 3 min after calcium addition respectively. Data were obtained from at least 8 independent experiments (n = 27–50). All data are normalized to the initial values and are expressed as mean ± SEM. Means were compared using one-way or two-way ANOVA, *p < 0.05, **p < 0.01, ***p < 0.001, *posthoc* Bonferroni test.

Mitochondrial handling of Ca^2+^ entry through capacitative calcium channels has been shown to regulate SOCE activity by preventing Ca^2+^-dependent slow inactivation^17^,^18^,^19^,^20^. To investigate the role of mitochondrial Ca^2+^ uptake in modulation of SOCE in neuroblastoma cells, we studied the effect of MCU knockdown. Neuro-2a cells were transfected with plasmids containing *Mcu*-directed small hairpin RNA (shRNA) or non-target control sequence (scrambled) along with mCherry, to identify transfected cells^40^, and studied 72 hours later. This resulted in a drop of MCU protein levels to 56.2 ± 8.3% of control values (Fig. 2A). [Ca^2+^]_i_ signals evoked by ATP were the same in scrambled or *Mcu*-KD cells (Fig. 2B). However, Ca^2+^ uptake in mitochondria (Ca_mit_) in response to these signals, studied with 4mt-D3cpv, a FRET calcium indicator targeted to the mitochondrial matrix^41^, was quite different. Ca^2+^ uptake in *Mcu*-KD was much lower than in scrambled mitochondria (Fig. 2B).

We next studied the effects of MCU silencing on Ca^2+^ entry through SOCE channels. We used the genetically encoded calcium indicator Lyn-D3cpv which is targeted to the plasma membrane^41^ in order to study changes in Ca^2+^ at the plasma membrane boundary, i.e, the site of SOCE. Figure 2C,D show that Ca^2+^ inflow upon SOCE activation with thapsigargin was reduced in *Mcu*-KD cells compared with scrambled sequences.

To determine whether decreased SOCE in SH-SY5Y *GDAP1*-KD cells is due to an impairment in mitochondrial Ca^2+^ handling, we studied Ca^2+^ influx through SOCE channels in control and the *GDAP1*-KD clone G4 previously described^15^ in presence of 0.25 mM 2,4-dinitrophenol (DNP), which collapses the mitochondrial membrane potential and prevents mitochondrial Ca^2+^ uptake. In control pLKO cells, DNP exposure caused a decreased SOCE, which was reduced to the level of *GDAP1*-KD cell line (Fig. 2E,F). In contrast, DNP exposure did not affect the amplitude of SOCE in *GDAP1*-KD cells, a result consistent with the hypothesis that GDAP1 deficiency prevents appropriate positioning of mitochondria and adequate handling of SOCE-driven Ca^2+^ inflow, thereby causing a decrease in SOCE^15^. Interestingly, DNP treatment in human SH-SY5Y neuroblastoma cells caused a decrease in SOCE activity to 71.5 ± 2.8% of control values and MCU silencing in mouse N2a neuroblastoma cells resulted in a similar decrease to 79 ± 6% of the levels in control cells treated with scrambled MCU sequences. These results support that a failure to take up Ca^2+^ in mitochondria next to the sites of SOCE opening causes the early inactivation of SOCE in GDAP1 deficient cells.

### *GDAP1* silencing impairs SOCE-driven stimulation of respiration

We next tested the effect of *GDAP1*-KD on SOCE-stimulation of respiration. Figure 2G shows that OCR stimulation caused by Ca^2+^ addition after ER-Ca^2+^ mobilization by carbachol is clearly lower in *GDAP1*-KD than control pLKO cells. Ca^2+^-dependent stimulation of respiration 3 min after Ca^2+^ addition, a time at which the SOCE-induced increase in [Ca]_i_ levels off (Fig. 2C,E), was 27.9 ± 2.4% above the initial values in control cells and 16.0 ± 0.7% in GDAP1 deficient cells (Fig. 2G,H). Interestingly, the increase in respiration caused by carbachol addition in a Ca^2+^-free medium is also reduced in the GDAP1 deficient cells (Fig. 2G,I), a result explained by lower ER-Ca^2+^ levels observed in *GDAP1*-KD cells^22^.

### Clinical *GDAP1*-CMT mutations have different effects on SOCE activity

**Figure 3:**
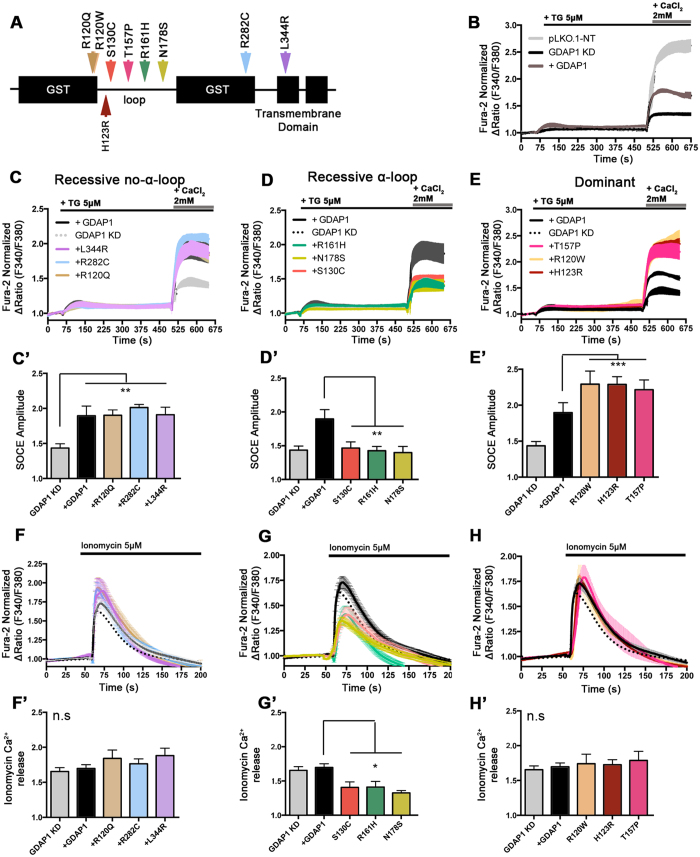
Pathological GDAP1 missense mutations show different effects on SOCE and ER-Ca^2+^ content depending on the mode of inheritance and their domain-location in the protein. (**A**) Schematic view of the GDAP1 missense mutations selected for the experiment. (**B**) SOCE rescue in *GDAP1*-KD cells (black line) transfected with pCAGIG-GDAP1 (brown line). The control pLKO cell line was transfected with pCAGIG empty vector and used as a control. Fura-2 [Ca^2+^]_i_ signals were measured upon addition of 5 μM TG in Ca^2+^-free medium and 2 mM Ca^2+^ where indicated. (**C**–**E**) SOCE in *GDAP1*-KD cells expressing recessive mutations located outside the protein-protein interaction domain α-loop (**C**), recessive mutations located inside the α-loop domain (**D**) and dominant mutations (**E**). Fura-2 [Ca^2+^]_i_ signals were measured upon addition of 5 μM TG in Ca^2+^-free medium and 2 mM Ca^2+^ where indicated. Quantification of SOCE amplitude as ΔRatio (F340/F380) is shown in (**C’**,**D’** and **E’**). (**F**–**H**) Comparison of ER-Ca^2+^ content by measuring ionomycin-induced Ca^2+^ release in the *GDAP1*-KD cell line or in the *GDAP1*-KD cell line overexpressing the indicated plasmids with recessive mutations outside **(F)** and inside the α-loop **(G)**, and dominant mutations **(H)**. Quantification of ionomycin-induced Ca^2+^ release amplitude as ΔRatio (F340/F380) is shown in (**F’**,**G’** and **H’**). Fura-2 [Ca^2+^]_i_ signals were measured upon addition 5 μM ionomycin in Ca^2+^-free medium. In all the experiments, more than 50 transfected cells were analyzed in 5 independent experiments. Data are normalized to the initial values and are expressed as mean ± SEM. Means were compared using one-way ANOVA test. *p < 0.05, **p < 0.01, ***p < 0.001, *posthoc* Bonferroni test.

GDAP1 has two GST domains separated by a region called α-loop, and a C-terminal transmembrane domain. We have previously found that the α-loop is the protein domain where caytaxin and RAB6B interact^15^. This suggests that mutations in the α-loop or even in the GST domains could affect the interaction with other protein partners, while mutations in the transmembrane domain may be related to failures in mitochondrial anchoring as proposed previously^42^. To address the role of these GDAP1 mutant proteins in restoring SOCE activity in *GDAP1*-KD cells, we selected a battery of dominant and recessive missense mutations located along the whole protein. For recessive mutations we selected p.R120Q, p.R282C and p.L344R as mutations outside the α-loop^43^,^44^,^45^ and p.S130C, p.R161H and p.N178S inside the α-loop^46^,^47^. We also selected p.R120W, p.H123R and p.T157P as a dominant mutations^6^,^48^,^49^ (Fig. 3A).

The effect related to each mutation was tested on SH-SY5Y *GDAP1*-KD cells after transfection with a bicistronic pCAGIG plasmid, which codified for the GDAP1 mutant and GFP, selecting for analysis only transfected cells. An empty vector (only GFP) and a vector with wild type GDAP1 were used as controls. SOCE was activated by emptying ER-Ca^2+^ with thapsigargin (TG) prior to activate SOCE with 2 mM CaCl_2_. As *GDAP1* silencing results in an increase in resting cytosolic Ca^2+ ^15, all responses were normalized to the basal level. The expression of empty vector does not alter SOCE activity (compare traces of control cell line pLKO with that of GDAP1, both expressing GFP (Fig. 3B)). Overexpression of WT GDAP1 protein does not recover SOCE activity at the level of control pLKO cells. Therefore, to address the effect of GDAP1 missense mutations we compared the responses of each mutant with cells overexpressing WT GDAP1 protein.

The GDAP1 effect on SOCE was different depending on the type of mutation. Recessive GDAP1 mutations located inside the α-loop were unable to compensate for the lack of GDAP1 in SOCE activity, indicating a complete loss of function. Neither p.S130C, p.R161H nor p.N178S could rescue the defect in SOCE and acted as the empty vector (Fig. 3D,D’). However, recessive mutations located outside loop, in TM (p.L344R) or GST domains (pR282C, R120Q) have the same effect on SOCE as GDAP1 (Fig. 3C,C’). In contrast, overexpression of dominant *GDAP1* missense mutations produced a completely different effect (Fig. 3E,E’). p.H123R and p.T157P, in the α-loop region, and p.R120W in a GST domain, all of them with an inherited dominant pattern, cause a significant increase in SOCE activity compared to wild type GDAP1, suggesting a gain of function of these mutations.

Taken together, the results suggest that recessive and dominant mutations in the α-loop and N-terminal flanking GST domain behave as lack of function or gain of function mutants respectively, maybe through their interference with protein-protein interactions within the α-loop domain. These interactions, possibly via caytaxin and RAB6B, are likely required for the adequate localization of mitochondria close to SOCE sites and their perturbation by mutations in GDAP1 is probably responsible for the inhibition of SOCE activity or stimulation of abnormal SOCE. On the other side, recessive mutations in other positions of the protein may act in a different way and do not show any impairment of SOCE activity. This is not surprising given the number of functions and interacting partners of GDAP1, and suggest that mutations in regions of the protein other than the α-loop lead to pathogenesis through impairment of their specific interactions. In agreement with this specificity, the p.R161H recessive mutation in the α-loop which we now show to impair SOCE activity did not have any effect on mitochondrial fission^9^.

As GDAP1 missense mutations within the α-loop domain reduce SOCE activity, and SOCE is related to filling of ER-Ca^2+^, these mutations may affect ER-Ca^2+^ levels. To address this possibility, we evaluated the Ca^2+^ transients induced by discharging ER-Ca^2+^ with ionomycin in a Ca^2+^-free media. Overexpression in *GDAP1*-KD cells of WT GDAP1, or any of the recessive missense mutant proteins out of the α-loop (Fig. 3F,F’) or any of the dominant mutant proteins (Fig. 3H,H’), resulted in similar Ca^2+^ transients obtained by discharge of ER-Ca^2+^. In contrast, transient expression of recessive GDAP1 mutant proteins with changes located inside the α-loop resulted in a decrease in the Ca^2+^ peak, suggesting that decreased SOCE activity results in a reduced capacity to refill the ER with Ca^2+^ (Fig. 3G,G’).

### Impaired mitochondrial localization at the suplasmalemmal domain in GDAP1 mutants

Our results are consistent with a major role of mitochondria in controlling SOCE activity in neuroblastoma cells. We previously found that mitochondria relocate to the suplasmalemmal (SP) domain (defined as 0–2 μm from plasma membrane) after ER-Ca^2+^ depletion in control but not in *GDAP1*-silenced neuroblastoma cells^15^. In order to address the mechanisms whereby the different mutations affect SOCE activity, we have studied the localization of mitochondria in relation to SP microdomains in basal conditions and after ER-Ca^2+^ depletion in neuroblastoma cells expressing dominant or recessive mutations in the α-loop. A Orai1::CFP expression vector was used to mark of the plasma membrane while the c-*myc* epitope from *GDAP1-c-myc* expression plasmids served as marker for mitochondria in immunofluorescence assays. The mitochondrial fluorescence distribution between opposite plasma membranes within the SP and the central cell zones was analyzed (Supplementary Fig. S2).

**Figure 4:**
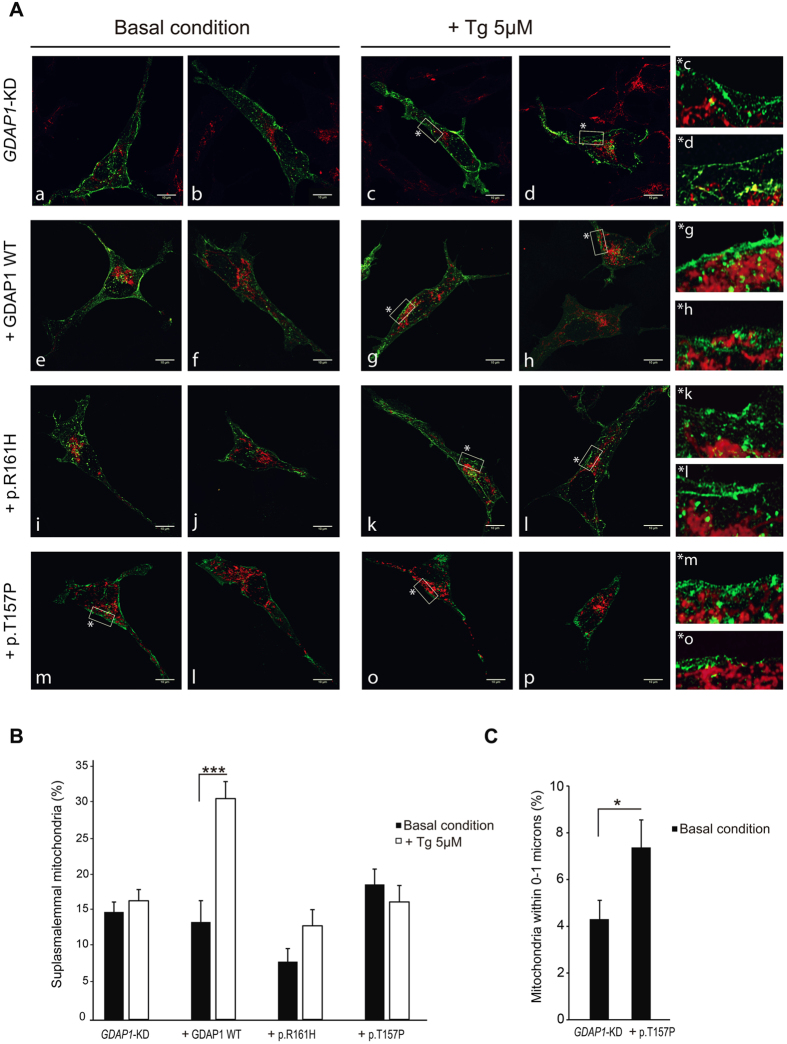
Mitochondrial network distribution in basal and SOCE activated conditions. **(A)** Confocal images of *GDAP1*-silenced neuroblastoma cells transfected with empty vector (mitochondria marked with α-βATPase, red signal), GDAP1 WT or mutant proteins (mitochondria marked with α-c-myc, red signal) and Orai1::CFP (green signal) in basal conditions and after ER-Ca^2+^ mobilization with 5 μM Tg in Ca^2+^-free medium. Cells were fixed after 10 min of vehicle or Tg treatment. Two images of each cell type and condition are shown; High resolution images of vehicle- (only in the case of dominant mutation) or Tg-treated cells are also shown on the right panel side. Bars indicate 10 μm. **(B)** Quantification of mitochondrial fluorescence distribution in basal conditions and after depletion of ER calcium stores with 5 μM Tg in Ca^2+^-free medium. Fluorescence distribution at the subplasmalemmal (SP) domains (defined as 2 μm underneath the plasma membrane) and the central zone (the space between the opposite subplasmalemmal domains) were calculated as indicated in Fig. S2. Fluorescence distribution in 7–9 cells per treatment and genotype is shown. (**C**) Quantification of mitochondrial fluorescence distribution obtained for mitochondria located 0–1 μm away from the plasma membrane. Results are expressed as mean ± SEM. Data were analyzed using two-way (**B**) or one way (**C**) ANOVA and *posthoc* Bonferroni tests. *p < 0.05, **p < 0.01, ***p < 0.001.

**Figure 5:**
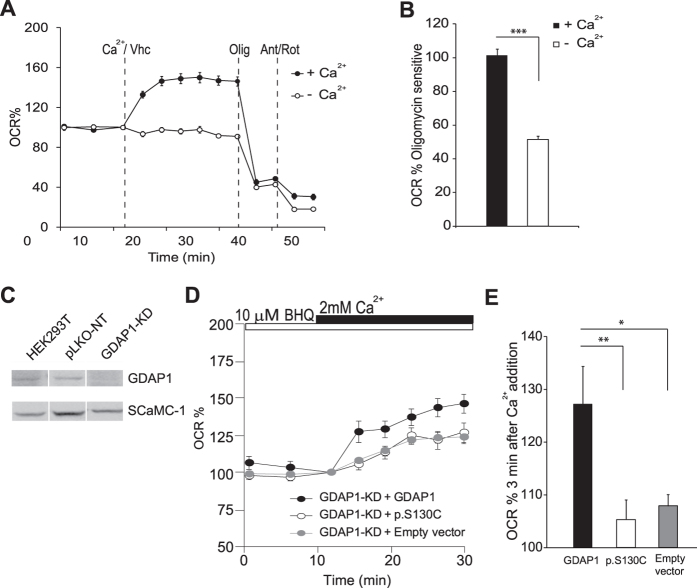
Recessive GDAP1 mutation p.S130C fails to recover SOCE stimulation of respiration in HEK293T *GDAP1*-KD cells. (**A**) SOCE-stimulation of respiration in HEK293T cell line. Oxygen consumption rate expressed as percentage of basal OCR in HEK293T cells treated with 10 μM BHQ in Ca^2+^-free medium, showing the sequential injection of 2 mM Ca^2+^ or vehicle (Vhc) and metabolic inhibitors. (**B**) Oligomycin sensitive OCR expressed as percentage of basal OCR in both conditions. The effect of calcium was significant (n = 11 from at least 3 independent experiments). (**C**) Analysis of GDAP1 levels in mitochondrial fraction by Western blot. Primary antibodies used were α-GDAP1 and α-SCaMC-1 as a control. The experiment was repeated twice with similar results. (**D**) SOCE stimulation respiration in HEK293T *GDAP1*-KD expressing WT GDAP1 protein, recessive GDAP1 mutant p.S130C or empty vector. Oxygen consumption rate expressed as percentage of basal OCR in cells treated with 10 μM BHQ in Ca^2+^-free medium. (**E**) Quantification of % OCR 3 min after calcium addition. Data were obtained from at least 4 independent experiments (n = 10–20). All data are expressed as mean ± SEM. Means were compared using one-way ANOVA test. *p < 0.05, **p < 0.01, ***p < 0.001, *posthoc* Bonferroni test.

As shown previously^15^, after ER-Ca^2+^ mobilization, mitochondria from *GDAP1*-silenced cells expressing an empty vector fail to localize at SP, resulting in a similar mitochondrial distribution in basal and SOCE-activation conditions (Fig. 4A,B). Re-expression of WT GDAP1 protein allowed mitochondria to be positioned in SP after ER-Ca^2+^ depletion (Figs 4A,g,h and 5B), while overexpression of the recessive mutation p.R161H had an effect similar to silencing of GDAP1, no relocation of mitochondria to SP (Fig. 4A,B). Mitochondria from cells overexpressing the dominant mutation do not relocalize to SP after ER-Ca^2+^ depletion (Fig. 4B) but show a surprisingly higher percentage of mitochondria close to SP under basal conditions (Fig. 4A,m). This difference is more pronounced in the interval from 0 to 1 μm from the plasma membrane (Fig. 4C), and suggests that this abnormal mitochondrial localization, closer to plasma membrane, may be associated with the higher SOCE activity caused by the dominant mutant.

### A GDAP1 variant in the α-loop domain fails to restore SOCE-stimulated respiration in *GDAP1*-silenced cells

To study the effect of pathogenic GDAP1-CMT mutations on SOCE-driven stimulation of respiration, neuroblastoma cells silenced for GDAP1 and expressing the different mutants were sorted out to obtain a pure preparation of mutant cells. However, the yields obtained were very low and all attempts towards a successful sorting failed. Therefore, we generated a stable *GDAP1*-silenced HEK293T cell line (*GDAP1*-KD) with about 70% reduction in GDAP1 protein levels and a control cell line expressing the empty vector (pLKO-NT) (Fig. 5C). To determine the effect of recessive GDAP1 mutations on SOCE-stimulated respiration in GDAP1-KD HEK293T cells, we expressed GDAP1 p.S130C variant, together with wild type GDAP1 or empty vector as controls. The percentage of cells expressing the vector with different constructions (judged by GFP fluorescence) was about 90%, and therefore, adequate for studies of respiration.

SOCE was activated by emptying ER-Ca^2+^ with 2,5-di-tert-butylhydroquinone (BHQ)^50^ in a Ca^2+^-free medium followed by 2 mM CaCl_2_ addition which resulted in a substantial SOCE-driven stimulation of mitochondrial respiration (Fig. 5A,B). Re-expression of WT GDAP1 protein increased SOCE-driven OCR in the *GDAP1*-KD cells while the p.S130C variant failed to restore the SOCE-driven stimulation in respiration. In fact, SOCE-driven respiration was not different from that of *GDAP1*-KD cells transfected with empty vector (Fig. 5D,E). These results suggest that the effects of recessive mutations in the α-loop domain on the SOCE activity could cause a reduced SOCE-driven stimulation of mitochondrial respiration.

## Concluding Remarks

Store-operated calcium channels are an essential pathway for calcium signaling in excitable or non-excitable cells, and mitochondria play an important role in SOCE modulation^51^,^52^. The present study has addressed the cause and functional consequences of the decrease in SOCE activity caused by GDAP1 deficiency in neuroblastoma cells. We do not assume any particular molecular composition of SOCE in these cells, and rather agree with a broad definition^53^, comprising currents with high (I_CRAC_) or low Ca^2+^ selectivity or even nonselective such as I_SOC_^54^, composed by TRP channels, particularly the TRPC subfamily. Thus, homo or heteromeric combinations of different channel subunits involving TRPCs and/or Orai interacting with STIM1 in the ER would confer them store operated properties. We show that mitochondrial Ca^2+^ uptake regulates SOCE activity in neuroblastoma cells, presumably by preventing its Ca^2+^-dependent inactivation, and that a failure to take up Ca^2+^ in mitochondria next to the sites of SOCE channels causes suppression of SOCE in GDAP1 deficient cells. We find a new function of SOCE, that of stimulation of respiration in neural cells. The upregulation of respiration is blocked in the presence of BAPTA, showing that Ca^2+^ signaling itself through regulation of mitochondrial respiration is required to couple respiration to SOCE activity. Consistently, upregulation of respiration by SOCE activation is impaired by GDAP1 deficiency. The release of ER Ca^2+^ also stimulates mitochondrial respiration and this stimulation decreases in GDAP1 deficient cells due to their lower ER-Ca^2+^ levels associated with the impairment of SOCE. Coupling stimulated respiration to ER-Ca^2+^ mobilization and SOCE-driven Ca^2+^ signals may involve Ca^2+^ actions on the external side of the inner mitochondrial membrane, where the Ca^2+^ binding domains of Aralar/AGC1 or the ATP-Mg/Pi carrier are located, and/or in the matrix, where Ca^2+^-regulation of mitochondrial dehydrogenases is known to take place^35^,^55^. Establishing which of these mechanisms is involved will aid in identifying potential targets in GDAP1 deficiency.

Interestingly, we find that a number of missense clinical-CMT *GDAP1* mutations also affect SOCE and phenocopy GDAP1 deficiency. These are the recessive mutations located in the α-loop domain involved in protein-protein interaction. The analysis of mitochondrial distribution in cells carrying these recessive mutations has now shown a failure to relocate mitochondria close to plasma membrane under SOCE-activation conditions, and suggest this failure as a likely cause for SOCE impairment. However, neither dominant mutations nor recessive mutations out of the α-loop affect SOCE in the way GDAP1 deficiency does. Interestingly, we have now observed that cells carrying the dominant mutations have an increase in SOCE amplitude, which might be related with a bias in mitochondrial distribution towards the plasma membrane under basal conditions, particularly the region within 1μm of the membrane. This bias may be due to an increased interaction of the dominant mutations with RAB6B and caytaxin^15^, and may explain an early prevention of SOCE inactivation at the plasma membrane by resident mitochondria. An increase in neuronal SOCE activity has been also shown to be detrimental to striatal neurons in a mouse model of Huntington’s disease^56^.

It is relevant to point out that the recessive forms of CMT involving *GDAP1* mutations are severe with an early onset and two of four recessive mutations associated with more severe phenotype (p.P153L and p.R161H) are located in the α-loop domain^57^. The cellular mechanism underlying the pathology of CMT caused by GDAP1 deficiency and by recessive mutations located in the α-loop domain may relate to a Ca^2+^-dependent bioenergetics failure along with abnormal mitochondrial distribution.

## Materials and Methods

### Cell lines cultures

Neuroblastoma control SH-SY5Y pLKO, *GDAP1*-KD cells previously described^15^ and Neuro-2a (N2a) were grown in DMEM-F12 (Gibco, Invitrogen, Carlsbad, CA) with 10% Fetal bovine serum (FBS), 2 mM L-glutamine and 100 mg/ml penicillin–streptomycin, at 37 °C with 5% CO_2_ and 2 μg/ml puromycin to maintain the selection. HEK293T cells were grown in DMEM with 10% FBS, 2 mM L-glutamine, nonessential amino acids and 100 mg/ml penicillin–streptomycin, at 37 °C with 5% CO_2_ and for stable *GDAP1*-silenced HEK293T cell clones 2 μg/ml puromycin was added to maintain the selection. The cultures were maintained at 37 °C in a humidified atmosphere of 5% CO_2_.

### Silencing *GDAP1* in HEK293T cell line

For the generation of a stable *GDAP1*-silenced HEK293T cell line, cells were transfected using Lipofectamine 2000 with the pLKO.1 vector (MISSION^®^ shRNA Plasmid DNA, Sigma-Aldrich) containing a hairpin sequence against *GDAP1* or a non-target control vector (pLKO-NT), containing 5 bp mismatches within the shRNA^22^. Upon selection with 2 μg/ml puromycin, clonal cell lines were obtained by limited dilution and tested for *GDAP1* silencing by Western Blot. HEK293T clones were cultured in 100 mm Petri dishes and were removed when cells were 90% confluent. Cells were collected with a scrapper into a homogenization buffer (250 mM Sucrose, 20 mM Hepes, 10 mM KCl, 1.5 mM MgCl_2_, 1 mM EDTA, 1 mM EGTA, 1 mM DTT, Complete protease inhibitor cocktail mini-EDTA free, (Roche, Mannheim, Germany); and adjusted to pH 7, 4) and were homogenized following differential centrifugation steps to separate different organelles. 40 μg of proteins of mitochondria-enriched fractions were separated by SDS-PAGE and transferred to nitrocellulose membranes. Primary antibodies used were α-GDAP1 (1:1000) mouse polyclonal (Abnova, Taipei, Taiwan) and α-SCaMC-1 (1:5000) rabbit polyclonal^58^ as mitochondrial control.

### Generation of *GDAP1* and missense *GDAP1* mutation vectors

We used the bicistronic pCAGIG expression vector for the simultaneous expression of *GDAP1* (wt or mutant form) and *GFP*. The pCAGIG-GDAP1 WT and p.R120W were previously described^22^. To produce the pCAGIG-GDAP1 p.R120Q, p.T157P and p.R282C plasmids, the GDAP1 mutant construct was obtained by restriction digestion respectively from the pCMV-Myc-GDAP1 p.R120Q, p.T157P and p.R282C^15^, and subcloned into the EcoRI-NotI sites of the pCAGIG plasmid. The GDAP1 p.H123R, p.S130C, p.N178S, p.R161H and p.L344R were obtained by PCR-based site-directed mutagenesis (Agilent Technologies, Santa Clara, CA, USA) by using the primers described in Appendix Table S1. The sequences of the constructs were confirmed by Sanger sequencing in an ABI Prism 3130xl autoanalyser (Applied Biosystems, Foster City, CA, USA). We used pCMV-myc plasmids for expression of *GDAP1* WT or mutant forms in immunofluorescence assays, previously described^15^.

### Measurement of cytosolic Ca^2+^ signals

Cytosolic calcium imaging with Fura-2 was performed as described by ref. 59. Cells were plated at 7.5 × 10^4^ cells/well onto 12 mm round coverslips. After 24 h, cells were loaded with Fura-2AM by incubation in 15 mM D-glucose Ca^2+^-free HCSS with 5 μM Fura-2AM and 50 μM pluronic F.127 acid (Both from Molecular Probes, Invitrogen, Carlsbad, CA, USA), for 30 min at 37 °C, and rinsed with HCSS 2 mM CaCl_2_, for 30 min. Fluorescence (emission 510 nm) ratio of Ca^2+^-free (F380) to Ca^2+^-bound probe (F340) was analyzed using Aquacosmos 2.5 software (Hamamatsu) and Metafluor for Leica developed by Metamorph (Universal Imaging). Regions of interest (ROIs) were selected covering single cells. In *GDAP1* mutations experiments, SH SY5Y cells were transiently transfected with the bicistronic pCAGIG plasmids using Lipofectamine 2000 (Invitrogen, Carlsbad, CA, USA) 24 hours before the experiment. Cells transfected with empty vectors were used as controls. In MCU silencing experiments, N2a cells were transfected 72 hours before with scrambled (shScr) or *Mcu*-directed (shMCU) containing vectors, kindly provided by Hilmar Bading and Giles E. Hardingham, which co-expressed mCherry protein^40^. MCU levels were tested by Western Blot, using α-MCU (1:500) rabbit polyclonal (Sigma-Aldrich, St. Louis, MO, USA) and mitochondrial α-βATPase (1:5000) rabbit polyclonal^60^, as a control. SOCE analysis was performed using a standard protocol, ER-Ca^2+^ was depleted using 5 μM of Thapsigargin (Alomone Labs, Jerusalem, Israel) or 250 μM carbachol (Sigma-Aldrich, St. Louis, MO, USA) in Ca^2+^-free HCSS media and SOCE was induced with 2 mM of CaCl_2_. ER-Ca^2+^ content analysis was performed after adding 5 μM of ionophores Br-A23187 or ionomycin (both from Sigma-Aldrich, St. Louis, MO, USA) in HCSS Ca^2+^-free media with 1 mM EGTA.

### Measurement of plasma membrane and mitochondrial Ca^2+^ signals

To image plasma membrane and mitochondrial Ca^2+^ levels, N2a cells were plated at a density of 4 × 10^4^ cells/well onto 4-wells Lab-Tek chamber slides (Nunc, Roskilde, Denmark) and co-transfected using Lipofectamine 2000 (Invitrogen, Carlsbad, CA, USA) 72 h prior to the experiment with pcDNA-lynD3cpv (Addgene^41^) or pcDNA-4mtD3cpv (Addgene^41^) encoding, respectively, for plasma membrane and mitochondrial targeted Ca^2+^ FRET probes, and shScr or shMCU. Only mCherry positive neurons were selected for the experiments. Experiments were performed in HCSS containing 15 mM glucose. Cells were excited for 100 ms at 436/20 mm and the emitted fluorescence was collected through a dual-pass dichroic CFP-YFP (440/500 nm and 510/600 nm alternatively at 480/40 nm (CFP) and 535/30 nm (YFP)). Images were collected every 5 s using a filter wheel (Lambda 10-2, Sutter Instruments; all filters purchased from Chroma) and recorded by a Hamamatsu C9100–02 camera mounted on an Axiovert 200M inverted microscope equipped with a 63X/1.4 oil Plan-Apochromat Ph3 objective. ROIs were selected on somas and neurites areas respectively and single-cell fluorescence recordings were analyzed using MetaMorph (Universal Imaging) and ImageJ (NIH).

### Measurement of cellular oxygen consumption

Cellular oxygen consumption rate (OCR) was measured using a Seahorse XF24 Extracellular Flux Analyzer (Seahorse Bioscience)^61^. Neuroblastoma SH-SY5Y and HEK293T cells were plated in XF24 V7 cell culture at 1.5 × 10^5^ cells/well and incubated for 24 h in a 37 °C, 5% CO_2_ incubator in culture medium. Cells were equilibrated with bicarbonate-free low-buffered DMEM medium (without pyruvate, lactate, glucose, glutamine, and Ca^2+^) supplemented with 15 mM glucose and 2 mM CaCl_2_ or 100 μM EGTA in conditions of ±Ca^2+^, for 1 h immediately before extracellular flux assay. Drugs were prepared in the same medium and were injected from the reagent ports automatically to the wells at the times indicated. Bioenergetics characterization of culture cells was determined through sequential addition of 6 μM oligomycin, 0.25 mM 2,4- dinitrophenol (DNP), and 1 μM antimycin/1 μM rotenone (all from Sigma-Aldrich, St. Louis, MO, USA). This allowed determination of basal oxygen consumption, oxygen consumption linked to ATP synthesis (ATP), non-ATP linked oxygen consumption (leak), maximal uncoupled respiration (MUR), and non-mitochondrial oxygen consumption (for review see ref. 62). To measure SOCE, first ER was emptying by adding 250 μM carbachol or 10 μM 2,5-di-tert-butylhydroquinone (BHQ) (Sigma-Aldrich, St. Louis, MO, USA), an inhibitor of SERCA, the ER-Ca^2+−^ ATPase (preincubated 1 h before the experiment). Then, SOCE activity was triggered by adding 2 mM CaCl_2_. Finally, oligomycin and antimycin/rotenone were used to calibrate the respiration. When required, BAPTA-AM (Sigma-Aldrich, St. Louis, MO, USA) loading was performed in Ca^2+^-free DMEM during 30 min before the experiment. For expressing *GDAP1* mutant forms, *GDAP1*-KD HEK293T cells were cultured in 100 mm Petri dishes and were transfected using the calcium phosphate method when cells were 70–75% confluent. 24 h after transfection, cells were seeded in a XF24 V7 cell culture plate and incubated for 24 h in a 37 °C, 5% CO_2_ incubator.

### Immunofluorescence assays and mitochondrial distribution analysis

Neuroblastoma SH-SY5Y cells were fixed with 2% paraformaldehyde (PFA) for 10 min and 4% PFA for 10 min and immunodetection was performed as described^58^. For mitochondrial detection, a mouse monoclonal α-c-myc antibody (Sigma-Aldrich, St. Louis, MO, USA) was used in GDAP1-transfected cells and a mouse monoclonal α-βATPase (Sigma-Aldrich, St. Louis, MO, USA) for transfected with empty vector. As secondary antibody a goat anti-mouse coupled to Alexa Fluor 555 (ThermoFisher, Waltham, MA, USA) was used. To address cytoplasmic mitochondrial network and its relation with plasma membrane during SOCE activation, Orai1::CFP (Addgene^63^) was used as plasma membrane marker. Images were taken using the Confocal LSM710 laser scanning microscope from Zeiss. Cells were excited at 458 nm for Orai1::CFP and at 561 nm for Alexa Fluor 555, and the emitted fluorescence was collected between 461 and 512 nm for CFP and 562–630 nm for Alexa 555. Fifteen-twelve images with 0.12 μm between each were acquired. Images were deconvolved with Huygens software (Scientific Volume Imaging) and analysis was done using ImageJ software (developed by NIH).

## Additional Information

**How to cite this article**: González-Sánchez, P. *et al*. CMT-linked loss-of-function mutations in *GDAP1* impair store-operated Ca^2+^ entry-stimulated respiration. *Sci. Rep.* **7**, 42993; doi: 10.1038/srep42993 (2017).

**Publisher's note:** Springer Nature remains neutral with regard to jurisdictional claims in published maps and institutional affiliations.

## Supplementary Material

Supplementary Information
