# Supplementary material for: CMT-linked loss-of-function mutations in GDAP1 impair store-operated Ca2+ entry-stimulated respiration
Source: Sci Rep. 2017 Feb 21;7:42993. doi: 10.1038/srep42993 (PMC5318958; doi:10.1038/srep42993)

Supplementary information

CMT-linked loss-of-function mutations in *GDAP1* impair store-operated Ca^{2+} entry-stimulated respiration

Paloma González-Sánchez[1,2,3†], David Pla-Martín[2,4†], Paula Martínez-Valero[1,2,3†], Carlos B Rueda[1,2,3], Eduardo Calpena[2,4], Araceli del Arco[2,3,5], Francesc Palau[2,4,6,7] *, Jorgina Satrústegui[1,2,3]*

[1]Departamento de Biología Molecular, Centro de Biología Molecular Severo Ochoa, Consejo Superior de Investigaciones Científicas–Universidad Autónoma de Madrid (CSIC-UAM), Madrid, 28049, Spain, [2]Centro de Investigación Biomédica en Red de Enfermedades Raras (CIBERER) Madrid, 28029, Spain, [3]Instituto de Investigación Sanitaria Fundación Jiménez Díaz, IIS-FJD, Madrid, 28040, Spain, [4]Program in Rare and Genetic Diseases and IBV/CSIC Associated Unit, Centro de Investigación Príncipe Felipe, Valencia, 46012, Spain, [5]Facultad de Ciencias Ambientales y Bioquímica, Universidad de Castilla la Mancha, Toledo, 45071, Spain, [6]Institut de Recerca Sant Joan de Déu and Hospotal Sant Joan de Déu, Barcelona 08950, Spain, [7]Pedriatrics Division University of Barcelona School of Medicine, Barcelona, Spain

Supplementary Table and Figures

Supplementary table S1. Primers used to generate GDAP1 mutants by PCR-based site-directed mutagenesis.

**GDAP1**	Forward	Reverse
p.H123R	ACCCACGGGTACAACGTTACCGAGAGCTG	AGCAGCTCTCGGTAACGTTGTACCCGTGG
p.S130C	GAGAGCTGCTTGACTGCTTGCCAATGGATGC	GCATCCATTGGCAAGCAGTCAAGCAGCTCTC
p.N178S	TTCTTGTAAATCTGGGCTTTCTTCAGCAAGTTTCTTCAGCTC	GAGCTGAAGAAACTTGCTGAAGAAAGCCCAGATTTACAAGAA
p.R161H	TTATGCAACTACAAGGATTCATAGCCAAATTGGAAACACAGAGTC	GACTCTGTGTTTCCAATTTGGCTATGAATCCTTGTAGTTGCATAA
p.L344R	CTTTTCAGAAAGAGGCGTGGCAGCATGAT	GCTAATATCATGCTGCCAGGCCTCTTTCTG

Supplementary Figure S1.

Mitochondrial distribution in subplasmalemal (SP) and central regions. To determine mitochondrial fluorescence profile, a projection of Z-stacks was done for each cell, removing the two upper and lower planes. For every cell 6-9 lines of different lengths between opposite plasma membrane covering the majority of mitochondrial network and avoiding the nucleus were drawn (A). Line length was between 8 – 18µm, depending on cell morphology. Cells and lines were chosen so that, for each condition, the number of lines of each length was similar. Lines were parallel and with a separation of 1,5 µm. Then, the fluorescence profile was calculated in the mitochondrial images (B) using ImageJ software. Mitochondrial fluorescence distribution in SP domains (defined as 2 µm underneath the plasma membrane) and the central zone (the space between the opposite SP domains) was calculated as percent of total fluorescence in each profile, using MATLAB software.

A B

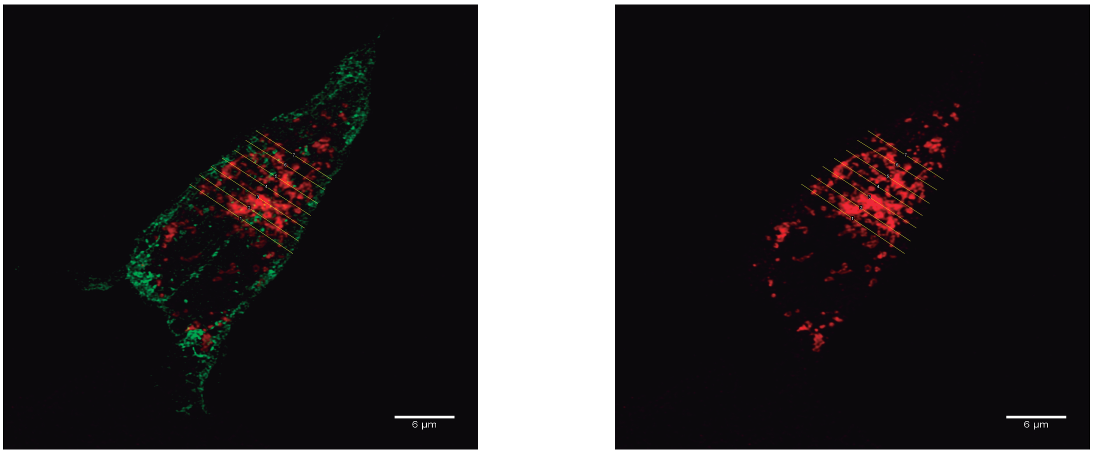

Supplementary Figure S2.

Mitochondrial Ca2+ uptake during SOCE and SOCE-stimulation of respiration is reduced in GDAP1-KD cells. Uncropped western blot images corresponding to Figure 2(A).

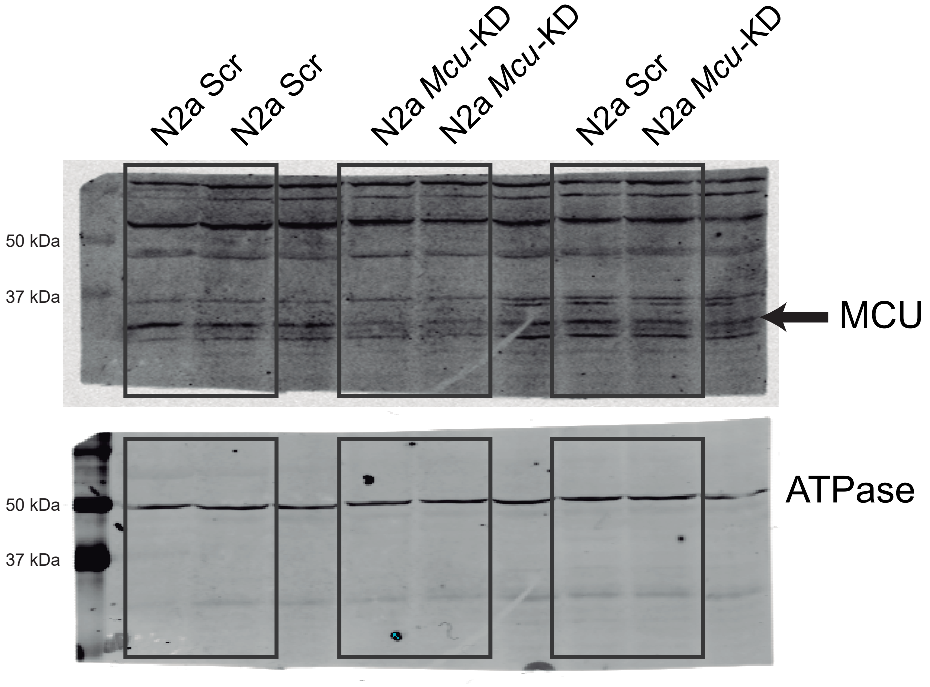

Supplementary Figure S3.

Recessive GDAP1 mutation p.S130C fails to recover SOCE stimulation of respiration in HEK293T GDAP1-KD cells. Uncropped western blot images corresponding to Figure 5(C).

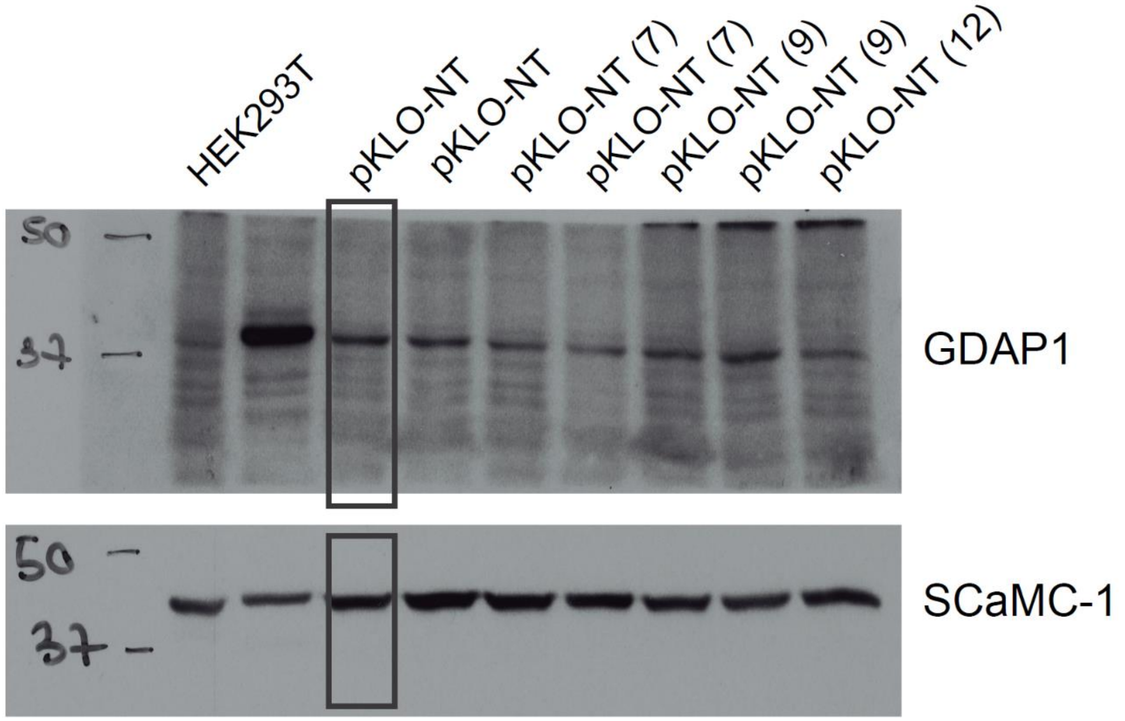

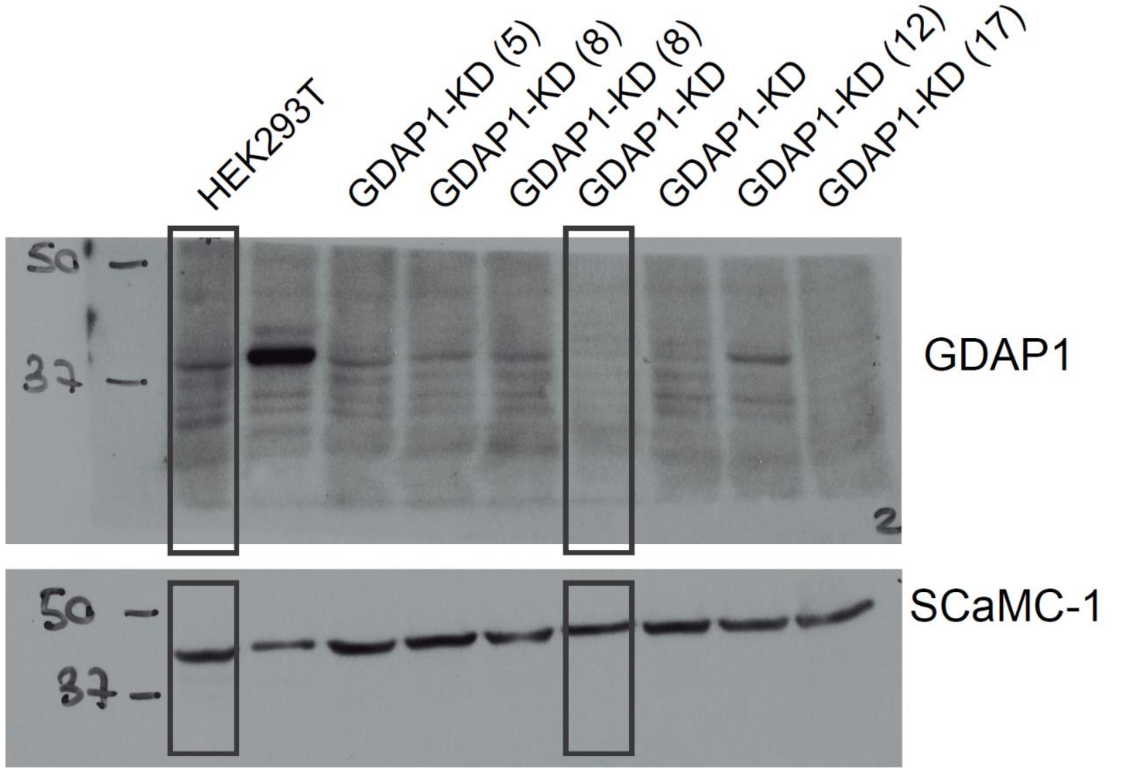

Supplement: Supplementary Information [file srep42993-s1.pdf]
